# Arketamine for cognitive impairment in psychiatric disorders

**DOI:** 10.1007/s00406-023-01570-5

**Published:** 2023-02-14

**Authors:** Kenji Hashimoto

**Affiliations:** Division of Clinical Neuroscience, Chiba University Center for Forensic Mental Health, 1-8-1 Inohana, Chiba, 260-8670 Japan

**Keywords:** Arketamine, Cognition, Esketamine, Gut microbiota, Ketamine

## Abstract

Cognitive impairment has been observed in patients with various psychiatric disorders, including schizophrenia, major depressive disorder (MDD), and bipolar disorder (BD). Although modern therapeutic drugs can improve certain symptoms (i.e., psychosis, depression) in these patients, these drugs have not been found to improve cognitive impairment. The *N*-methyl-D-aspartate receptor antagonist (*R,S*)-ketamine has attracted attention as a rapidly acting antidepressant. In addition to its robust antidepressant effects, (*R,S*)-ketamine has been suggested to improve cognitive impairment in patients with MDD and BD, despite causing cognitive impairment in healthy control subjects. (*R*,*S*)-ketamine is a racemic mixture of equal amounts of (*R*)-ketamine (or arketamine) and (*S*)-ketamine (or esketamine). Arketamine has been found to have more potent antidepressant-like actions than esketamine in rodents. Interestingly, arketamine, but not esketamine, has been suggested to improve phencyclidine-induced cognitive deficits in mice. Furthermore, arketamine has been suggested to ameliorate cognitive deficits in rodent offspring after maternal immune activation. In the current article, it is proposed that arketamine has therapeutic potential for treating cognitive impairment in patients with psychiatric disorders. Additionally, the potential role of the gut–microbiome–brain axis in cognitive impairment in psychiatric disorders is discussed.

## Introduction

**Fig. 1:**
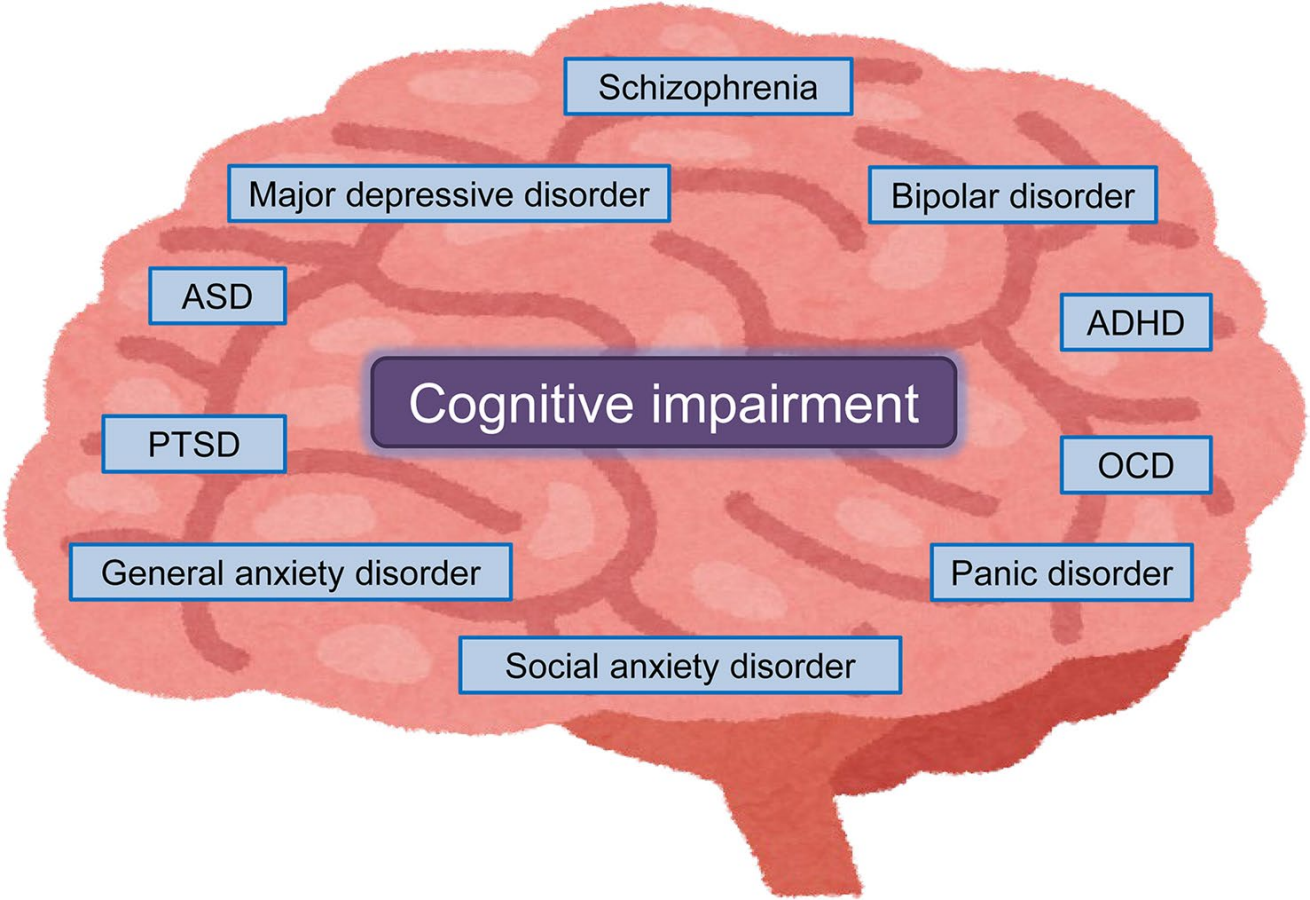
Cognitive impairment in patients with psychiatric disorders. ADHD: attention deficits hyperactivity disorder. ASD: autism spectrum disorder. OCD: obsessive–compulsive disorder. PTSD: post-traumatic stress disorder. Some elements of the figure were created using resources from www.irasutoya.com

**Fig. 2:**
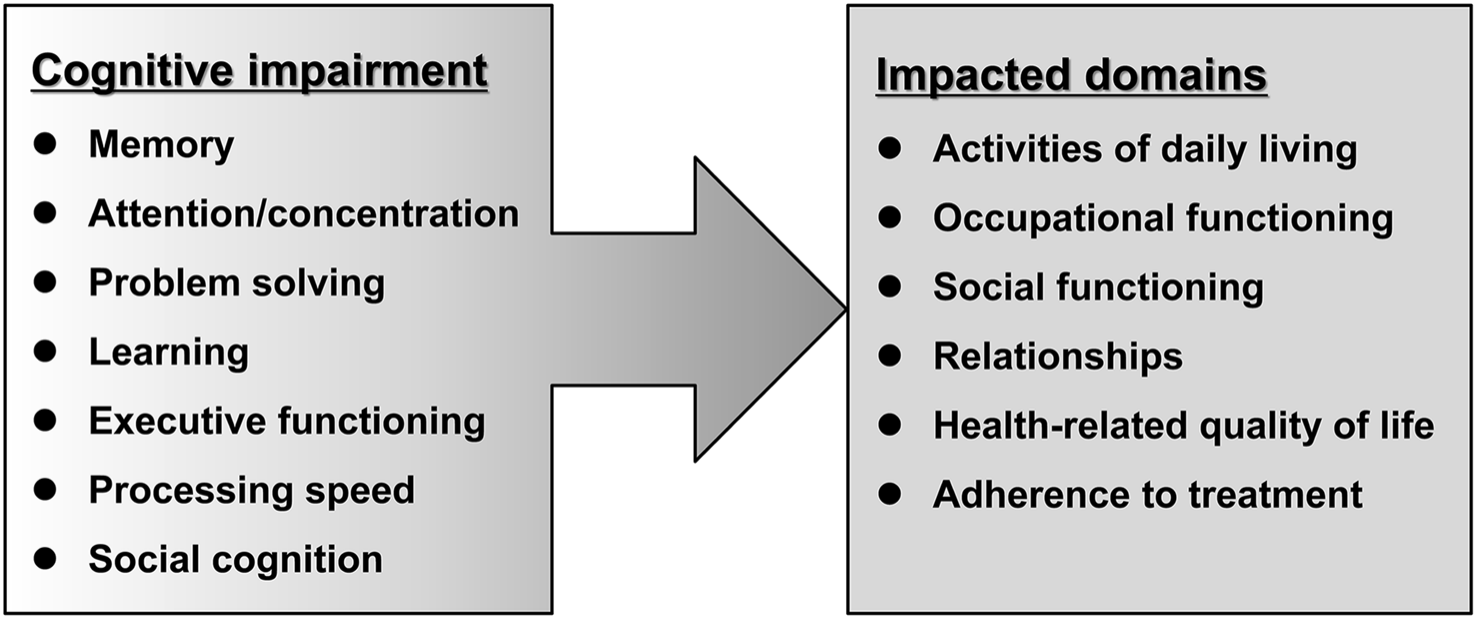
Impact of cognitive impairment in patients with schizophrenia. Cognitive functions affected in patients with schizophrenia include memory, attention/concentration, problem solving, learning, executive function, processing speed, and social cognition. Decline of cognitive functioning impacts the ability of individuals to carry out activities of daily living, occupational functioning, social functioning, relationships, health-related quality of life, and adherence to treatment. This figure was modified from reference [6]

Cognitive impairment is common in patients with various psychiatric disorders, including schizophrenia, major depressive disorder (MDD), bipolar disorder (BD), autism spectrum disorder (ASD), post-traumatic stress disorder (PTSD), attention deficit/hyperactivity disorder (ADHD), obsessive–compulsive disorder (OCD), panic disorder, generalized anxiety disorder, and social anxiety disorder (Fig. 1) [1–3]. Several batteries, such as the Brief Assessment of Cognition in Schizophrenia (BACS), MATRICS Consensus Cognitive Battery (MCCB), Cambridge Neuropsychological Test Automated Battery (CANTAB), the Cogstate battery, and the Repeatable Battery for the Assessment of Neuropsychological Status (RBANS) have been used to measure cognitive function in humans. Using the Cogstate battery, we previously reported that cognitive impairment in patients with schizophrenia was more severe than that in patients with MDD [4, 5]. The affected cognitive domains in patients with schizophrenia include memory, attention/concentration, problem solving, learning, executive function, processing speed, and social cognition. Furthermore, declines of these cognitive functions impact various domains, such as activities of daily living, occupational functioning, social functioning, relationships, health-related quality of life and adherence to treatment, resulting in increased direct and indirect costs associated with the treatment of schizophrenia (Fig. 2) [6]. A recent systematic review showed cognitive deficits in patients with MDD in the acute and remitted state [3]. Cognitive impairment of psychiatric disorders is not just a secondary consequence of perturbed affect, despite a close relationship between cognition and psychiatric symptoms. Although certain symptoms (i.e., psychosis, depression, anxiety) in patients with psychiatric disorders could be alleviated by the current therapeutic drugs, these drugs could not improve cognitive impairment [1]. Therefore, the development of novel therapeutic drugs for cognitive impairment is an unmet need [7–9].

**Fig. 3:**
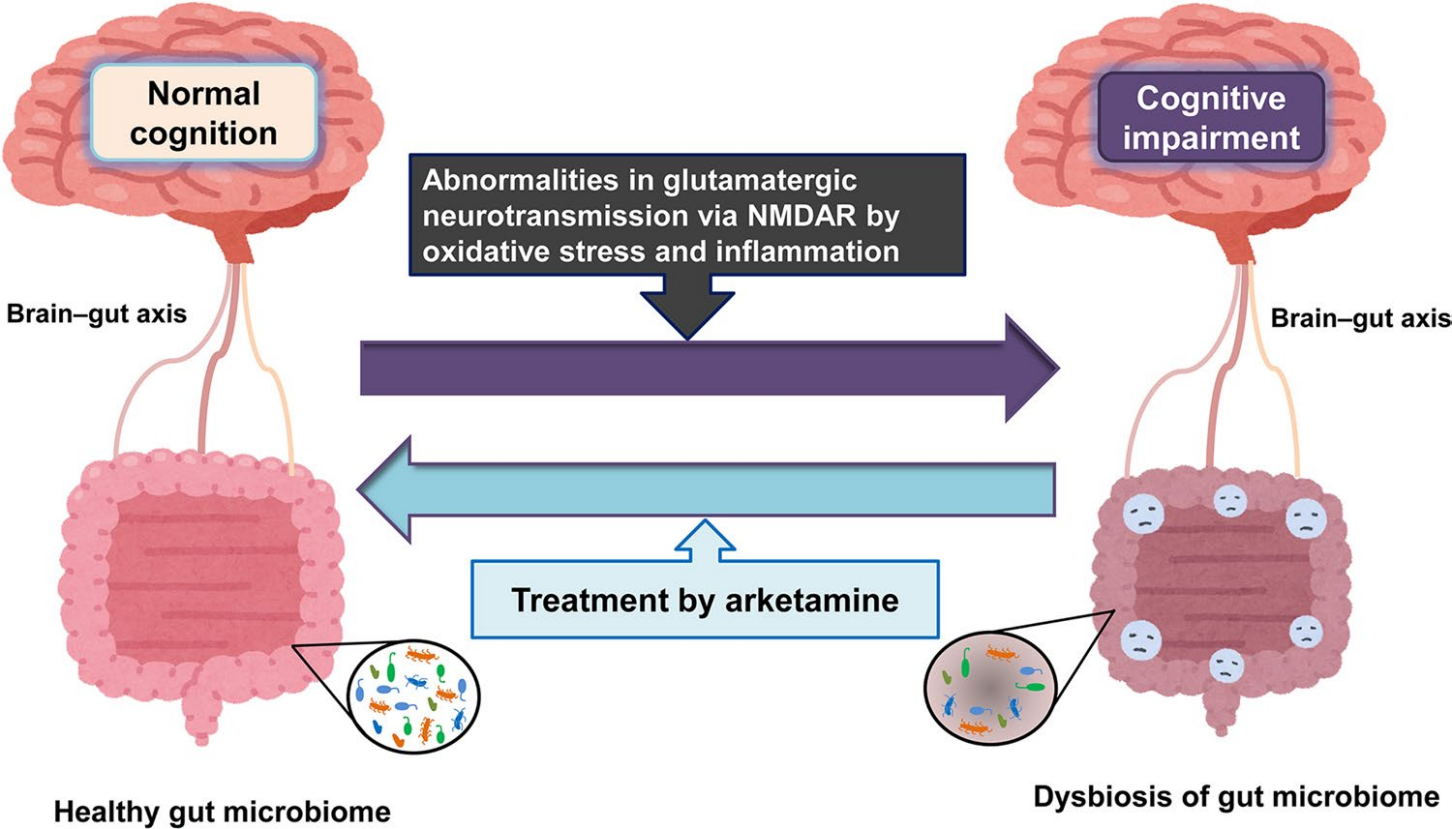
Therapeutic potential of arketamine in cognitive impairment of psychiatric disorders. Abnormalities in glutamatergic neurotransmission via the NMDAR by oxidative stress and inflammation may play a role in the cognitive impairment and dysbiosis of gut microbiota in patients with psychiatric disorders. Accumulating evidence suggests that dysbiosis of gut microbiota may play a role in cognitive impairment in patients with psychiatric disorders. Therefore, it is likely that arketamine improves cognitive impairment in patients with psychiatric disorders through the gut–microbiota–brain axis. The figure was modified from references [36, 44]. Some elements of the figure were created using resources from www.irasutoya.com

Glutamatergic neurotransmission via the *N*-methyl-D-aspartate receptor (NMDAR) regulates synaptic plasticity, memory, and cognition. Abnormalities in glutamatergic neurotransmission via the NMDAR play a role in cognitive impairment of psychiatric disorders (Fig. 3). In addition to psychomimetic and dissociative symptoms, NMDAR antagonists such as phencyclidine (PCP) and (*R,S*)-ketamine are known to cause cognitive impairment in rodents and humans [10–18]. There are several reports showing abnormalities in NMDAR-mediated amino acids (i.e., D-serine and L-serine) in patients with schizophrenia [19–22]. Recent mega-analysis of proton magnetic resonance spectroscopy (MRS) shows altered levels of glutamate in the brain from patients with schizophrenia [23]. Furthermore, abnormalities in NMDAR-mediated neurotransmission are implicated in mood disorders such as MDD and BD [24–29]. Meta-analyses of MRS studies showed altered levels of glutamate in the brain from patients with MDD or BD [30, 31]. Interestingly, there were significant correlations between cognitive functions and glutamate levels in the brain from first episode drug-naïve patients with MDD [32]. Collectively, it is likely that abnormalities in glutamatergic neurotransmission might play a role in the cognitive impairment in patients with psychiatric disorders (Fig. 3). Taken all together, previous research suggests that NMDAR could be a therapeutic target for cognitive impairment in patients with psychiatric disorders [14, 18, 33, 34].

It is widely recognized that (*R,S*)-ketamine causes cognitive impairment in healthy subjects [12, 13, 35]. However, increasing evidence suggests that (*R,S*)-ketamine may improve cognitive impairment in patients with mood disorders such as MDD and BD [36, 37]. In the current article, the author reviewed the therapeutic potential of (*R,S*)-ketamine and its enantiomer (*R*)-ketamine (or arketamine) for cognitive impairment in neuropsychiatric disorders. Furthermore, the author discussed the possible role of the gut–microbiota–brain axis in cognitive impairment of psychiatric disorders.

### Brief history of (*R*,*S*)-ketamine and its enantiomers

In 1962, (*R*,*S*)-ketamine was synthesized as an alternative short-acting anesthetic of PCP [38]. In 1970, (*R*,*S*)-ketamine was approved for use as an anesthetic in the United States of America (USA). In 1985, (*R*,*S*)-ketamine was included on the World Health Organization’s List of Essential Medicines [39]. (*R*,*S*)-ketamine is a racemic mixture of equal amounts of (*R*)-ketamine (or arketamine) and (*S*)-ketamine (or esketamine). Esketamine has greater affinity for the NMDAR than arketamine. Because the anesthetic effect of esketamine in human volunteers was found to be more potent than that of arketamine [40, 41], esketamine has been used as an anesthetic in the European Union (EU) and China.

In the research field of mood disorders, (*R*,*S*)-ketamine has attracted attention as a rapidly acting antidepressant [36, 42–46]. In 2000, Berman et al. [47] reported that a single intravenous infusion of (*R*,*S*)-ketamine (0.5 mg/kg) produced rapidly acting and sustained antidepressant effects in drug-free patients with MDD. Subsequent studies confirmed robust antidepressant and anti-suicidal effects of (*R*,*S*)-ketamine (0.5 mg/kg) in treatment-resistant patients with MDD or BD [48–52]. (*R*,*S*)-ketamine has been widely used as an off-label treatment in the USA and EU, despite the current lack of safety data [53–55]. In 2019 and 2020, a nasal spray containing esketamine produced by Johnson and Johnson was approved in the USA and EU for treatment-resistant patients with MDD and people at high risk of suicide. However, there are several concerns about the efficacy and approval of esketamine nasal spray [56, 57].

An increasing number of preclinical studies has suggested that arketamine has greater potency and longer lasting antidepressant-like effects than esketamine in rodent models of depression, although the affinity of arketamine at NMDAR is less potent than that of esketamine [58–66]. Behavioral and biological abnormalities in rodents (i.e., hyperactivity, prepulse inhibition, dopamine release from the synaptic terminal, abuse liability, parvalbumin (PV)-immunoreactivity in the prefrontal cortex (PFC), and heat shock protein HSP-70 expression in the retrosplenial cortex) after injection of arketamine in animals were reduced compared with those after (*R,S*)-ketamine or esketamine [59, 67–71].

An open-label pilot study in Brazil reported that a single infusion of arketamine (0.5 mg/kg) caused rapid and sustained antidepressant effects in treatment-resistant patients with MDD [72]. Importantly, side effects (i.e., psychotomimetic and dissociative effects) of arketamine (0.5 mg/kg) in treatment-resistant patients with MDD [72] are substantially less severe than those of esketamine (0.2 and 0.4 mg/kg, i.v.) [73]. Taken together, previous findings suggest that arketamine might provide a novel antidepressant without the side effects of (*R,S*)-ketamine and esketamine. A phase 2 study of arketamine (or PCN-101) in treatment-resistant patients with MDD is currently being conducted by Perception Neuroscience, Inc. (New York, USA) [39, 46, 74].

It is well known that non-ketamine NMDAR antagonists/modulators do not produce ketamine-like robust antidepressant actions in patients with MDD, suggesting that NMDAR might not play a major role in the antidepressant effects of (*R*,*S*)-ketamine in patients with depression [36, 42, 75–77]. However, the precise molecular and cellular mechanisms underlying the antidepressant effects of arketamine remain elusive [36, 39, 46, 78–80].

### Effects of (*R*,*S*)-ketamine and its enantiomers on cognition in healthy subjects

NMDAR antagonists such as PCP and ketamine are known to cause schizophrenia-like symptoms in healthy subjects, including cognitive impairment [12, 13]. In addition to positive and negative symptoms, intravenous administration of (*R,S*)-ketamine (0.5 mg/kg) produced cognitive impairments in healthy subjects [35]. Furthermore, intravenous administration of esketamine (0.1 mg/kg/min for 5 min and 0.006 mg/kg/min for 60 min) or (*R,S*)-ketamine (0.2 mg/kg/min for 5 min and 0.012 mg/kg/min for 60 min) to healthy subjects was found to produce significant psychopathological and neurocognitive impairment compared with placebo [81]. Interestingly, esketamine, but not (*R,S*)-ketamine, significantly increased the auditory alterations subscore of the five-dimensional questionnaire for the assessment of altered states of consciousness, suggesting that arketamine may have a potential protective effect against esketamine-induced psychotomimetic effects [81]. Furthermore, a single intranasal infusion of esketamine (84 mg) in healthy subjects caused a significant cognitive performance impairment at 40 min for all five Cogstate tests, although there were no changes between the esketamine group and the placebo group at 2, 4, or 6 h after infusion [82].

A recent meta-analysis showed acute impairment of cognition in healthy subjects after acute infusion of (*R,S*)-ketamine or esketamine [83]. Furthermore, verbal learning and memory are the functions most prominently affected in cognitive impairment caused by acute injection of (*R,S*)-ketamine or esketamine [83]. Thus, it is possible that (*R,S*)-ketamine and esketamine produce cognitive impairment in healthy subjects.

### Effects of (*R*,*S*)-ketamine on cognitive impairment in patients with MDD or BD

Clinical studies suggest that (*R,S*)-ketamine may improve cognitive impairment in patients with mood disorders. Six repeated infusions of (*R,S*)-ketamine (0.5 mg/kg) were found to ameliorate cognitive impairment (i.e., processing speed) in treatment-resistant patients with MDD or BD [84–86]. A systematic review revealed that (*R,S*)-ketamine infusion led to significant improvements in cognitive impairment in treatment-resistant patients with MDD, and (*R,S*)-ketamine did not worsen cognitive function in depressed patients [87]. Furthermore, the improvement in working memory may be predictive of the anti-suicidal ideation response to (*R,S*)-ketamine in treatment-resistant patients with MDD [88]. Repeated infusion of (*R,S*)-ketamine (0.5 mg/kg) caused significant improvement of working memory in MDD patients with PTSD [89]. Interestingly, depression symptom severity and processing speed performance in patients with MDD or BD partially mediated the improvements in suicidal ideation after repeated infusion of (*R,S*)-ketamine [90]. A recent systematic review indicated potential procognitive effects of subanesthetic doses of (*R,S*)-ketamine among patients with depression, although there is evidence for immediate altered cognitive dysfunction in healthy subjects [91]. In addition, precognitive effects of (*R,S*)-ketamine were pronounced in cognitive domains of executive function. A short course of repeated infusion of (*R,S*)-ketamine (0.5 mg/kg) produced significant improvements in several cognitive domains, including attention, working memory, verbal memory, and visuospatial memory in treatment-resistant patients with MDD [92]. Taken together, these findings suggest that (*R,S*)-ketamine has beneficial effects on cognitive impairment in depressed patients, although further studies with larger sample sizes are needed.

Patients with MDD or BD typically exhibit a range of negative beliefs, such as worthlessness, hopelessness, and pessimism, and these conditions are considered to be a major public mental health concern [93, 94]. A recent study demonstrated that infusion of (*R,S*)-ketamine (0.5 mg/kg) improved depressive symptoms in treatment-resistant patients with MDD, and that the improvement was associated with changes in belief-updating processes [95]. These recent data provide new insights into the cognitive mechanisms of action of (*R,S*)-ketamine in mood disorders.

In contrast, Ochs-Ross et al. [96, 97] reported that intranasal injection of esketamine did not induce any changes in cognitive function of MDD patients from baseline, indicating a lack of beneficial effects of esketamine nasal spray on cognitive impairment.

### Effects of ketamine enantiomers on PCP-induced cognitive deficits in rodents

It is well known that NMDAR antagonists such as PCP cause cognitive deficits in rodents. Using the novel object recognition test, we previously reported that repeated administration of PCP (10 mg/kg/day for 10 days) caused cognitive deficits in mice over a long period (more than 6 weeks), and that PCP-induced cognitive deficits could be improved by subsequent sub-chronic administration of clozapine, but not haloperidol [98]. Using the paradigm of PCP-induced cognitive deficits, we reported several candidates for cognitive impairment in psychiatric disorders [99–103].

We compared the effects of two ketamine enantiomers in a PCP-induced cognitive deficits model. Interestingly, PCP-induced cognitive deficits in mice were ameliorated after subsequent repeated intermittent administration of arketamine (10 mg/kg/day, twice weekly for 2 weeks), but not esketamine (10 mg/kg/day, twice weekly for 2 weeks) [104]. Western blot analysis showed decreased levels of brain-derived neurotrophic factor (BDNF) in the PFC and hippocampus of PCP-treated mice [104]. Furthermore, the beneficial effects of arketamine on cognitive deficits of PCP-treated mice were blocked by pretreatment with TrkB inhibitor ANA-12. These findings suggest that arketamine could ameliorate PCP-induced cognitive deficits via activation of BDNF-TrkB signaling in the brain [104]. Taken together, these findings suggest that arketamine could potentially provide a useful therapeutic drug for cognitive impairment in patients with schizophrenia (Fig. 3).

### Cognitive impairment in the prodromal state of psychosis and the potential of arketamine

Cognitive impairment has been shown in the prodromal stage of psychosis [105–109]. Findings from meta-analyses support neurocognitive dysfunction as a potential detection and prognostic biomarker in individuals at clinical high risk (CHR) for psychosis [106, 107]. Therefore, it is important to treat cognitive impairment in individuals at CHR for psychosis to block the conversion to psychosis.

Epidemiological data suggest that maternal immune activation (MIA), such as maternal infection, might be associated with the risk of neuropsychiatric disorders, such as schizophrenia and ASD in offspring [110, 111]. During the coronavirus disease 2019 (COVID-19) pandemic, an increasing number of pregnant women have become infected with COVID-19 worldwide, and MIA induced by COVID-19 infection has been suggested as a risk factor for schizophrenia and ASD [112–114]. A cohort study shows that severe acute respiratory syndrome-coronavirus-2 (SARS-CoV-2) exposure in utero may be associated with neurodevelopmental sequelae in some offspring [115].

Toll-like receptor-3 agonist polyriboinosinic–polyribocytidylic acid (poly[I:C]) has been used to establish a rodent model of MIA [116, 117]. Exposure of pregnant mice to poly(I:C) causes cognitive deficits in juvenile offspring [118–121], and these cognitive deficits in juvenile offspring appear to be similar to the prodromal stage of psychosis.

**Fig. 4:**
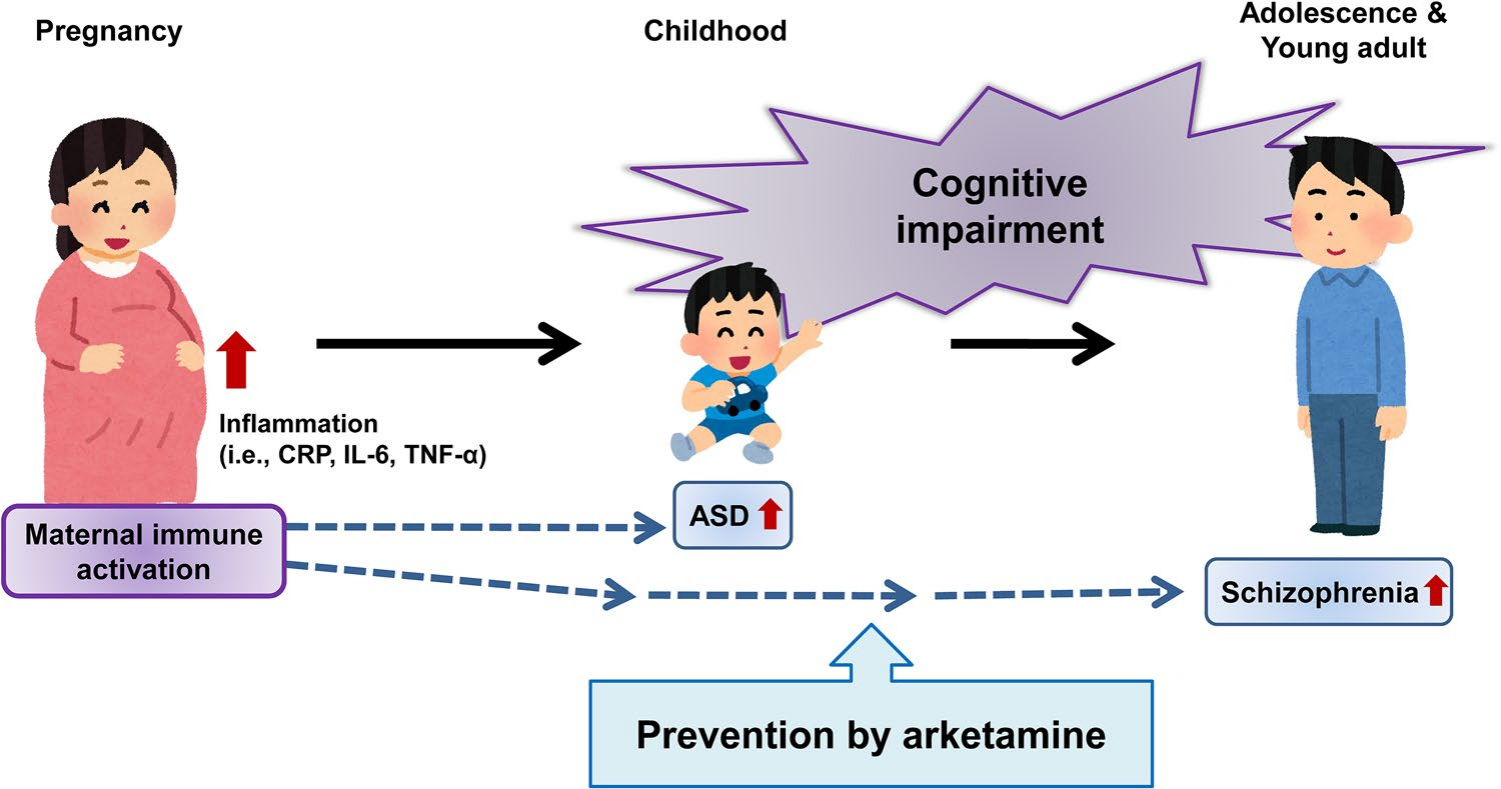
Therapeutic potential of arketamine in subjects at ultra-high risk (UHR) for psychosis. Maternal immune activation (MIA), such as that caused by maternal infection, causes inflammatory events in pregnant women, resulting in higher levels of inflammatory biomarkers (i.e., C-reactive protein [CRP], IL-6, and TNF-α) in the blood and tissues. Epidemiological data suggest that MIA can increase the risk of ASD and schizophrenia in offspring. Because subjects at UHR for psychosis have cognitive impairment as a prodromal symptom, early intervention using arketamine may block the onset of neuropsychiatric disorders in subjects at UHR of psychosis. This figure was modified from Fig. 3 in reference [113]. Some elements of the figure were created using resources from www.irasutoya.com

We investigated whether arketamine could prevent the development of psychosis-like phenotypes in adult offspring after MIA. We examined the effects of arketamine (10 mg/kg/day, twice weekly for 4 weeks) during juvenile and adolescent stages (P28–P56) on the development of cognitive deficits, loss of PV-immunoreactivity in the medial PFC (mPFC), and decreased dendritic spine density in the mPFC and hippocampus from adult offspring after prenatal poly(I:C) exposure [122]. Repeated intermittent administration of arketamine (10 mg/kg/day, twice weekly for 4 weeks) during juvenile and adolescent stages (P28–P56) significantly blocked the development of cognitive deficits, reduced PV-immunoreactivity in the prelimbic (PrL) of mPFC, and decreased dendritic spine density in the PrL of the mPFC, CA3, and dentate gyrus of the hippocampus from adult offspring after MIA. Furthermore, pretreatment with TrkB inhibitor ANA-12 significantly blocked the beneficial effects of arketamine on cognitive deficits of adult offspring after MIA [122]. These data suggest that repeated intermittent administration of arketamine during the juvenile and adolescent stages could prevent the development of psychosis in adult offspring after MIA through activation of BDNF-TrkB signaling. Therefore, it is possible that arketamine represents a useful prophylactic drug to prevent subsequent conversion from UHR to psychosis (Fig. 4).

### Dysbiosis of gut microbiota and cognitive impairment of psychiatric disorders

Accumulating evidence suggests the role of dysbiosis of gut microbiota in a variety of psychiatric disorders [123–126]. A narrative review shows that intervention of gut microbiota can improve cognitive or brain function, suggesting a role of gut microbiota in cognitive performance [127]. A recent population-based study of middle-aged adults demonstrated that microbial community composition on the basis of beta-diversity was associated with all cognitive measures in multivariable-adjusted analysis [128], suggesting a role of gut microbiota in cognitive decline with aging. It has also been suggested that dysbiosis of gut microbiota may play a role in cognitive impairment in patients with psychiatric disorders such as schizophrenia, MDD, and BD [128–132].

In addition to rapid antidepressant-like effects, arketamine, (*R,S*)-ketamine, and (S)-norketamine have been suggested to improve abnormal composition of gut microbiota in mice with depression-like behaviors [133–138]. Furthermore, arketamine could ameliorate abnormal composition of gut microbiota in mouse models of multiple sclerosis [139] and postmenopausal osteoporosis [140]. Considering the beneficial effects of arketamine on dysbiosis of gut microbiota, it is likely that arketamine may improve cognitive impairment in patients of psychiatric disorders through the gut–microbiota–brain axis [36, 42, 44, 125, 126]. Therefore, it is of interest to investigate whether arketamine can improve cognitive impairment and abnormal composition of gut microbiota in patients with psychiatric disorders.

## Conclusion and future directions

As stated in the introduction, cognitive impairment is shown in patients with a variety of psychiatric disorders. Neural mechanisms of cognitive impairment between schizophrenia and mood disorders such as MDD and BD may be different. However, a systematic review of MRS studies suggest that abnormal neurotransmission of glutamate and GABA (γ-aminobutyric acid) plays a role in cognitive impairment in patients with schizophrenia and mood disorders such as MDD and BD [141]. Given the role of glutamine–glutamate–GABA cycle in the brain [14, 16, 142], it is possible that abnormalities in the neurotransmission of glutamate and GABA may contribute to cognitive impairment in patients with psychiatric disorders such as schizophrenia and mood disorders. Nonetheless, further study is needed to ascertain the role of glutamate and GABA on cognitive impairment in patients with a variety of psychiatric disorders.

As discussed above, accumulating clinical data suggest that (*R,S*)-ketamine could improve cognitive impairment in patients with MDD or BD, although it causes cognitive impairment in healthy subjects. Preclinical data suggest that arketamine, but not esketamine, can improve PCP-induced cognitive deficits in rodents [104]. Furthermore, there is evidence that arketamine can ameliorate cognitive deficits in offspring after MIA through activation of BDNF-TrkB signaling [122]. Preclinical findings suggest that BDNF-TrkB signaling could play a role in the beneficial effects of arketamine in several animal models [36, 42–45, 66, 143–147]. However, the precise molecular and cellular mechanisms underlying the beneficial effects of arketamine remain elusive [36, 42–46, 148].

The COVID-19 pandemic began at the end of 2019, and continues to the present. The COVID-19 pandemic causes short-term and long-term mental health problems in survivors after SARS-CoV-2 infection [149–152]. A recent meta-analysis suggests that half of COVID-19 survivors have a high burden of either physical or mental sequelae (i.e., cognitive impairment) for up to at least 12 months [153]. Additionally, it may be useful to investigate whether arketamine can improve long-term mental sequelae in COVID-19 survivors.

Clinical trials of arketamine in healthy subjects and treatment-resistant patients with MDD are currently being conducted by several pharmaceutical companies, including Perception Neuroscience Inc. (USA), Otsuka Pharmaceutical Co., Ltd. (Japan), Jiangsu HengRui Medicine Co., Ltd. (China), and Jiangsu Enhua Pharmaceutical Co., Ltd. (China) [46]. Given the detrimental effects of cognitive impairment in patients with psychiatric disorders [1], it is of great interest to investigate whether arketamine could improve cognitive impairment in a number of psychiatric disorders, including schizophrenia, MDD, and BD. Finally, future clinical studies are needed to ascertain the efficacy of arketamine on cognitive impairment in patients with psychiatric disorders.

## Data Availability

This article does not contain any original data.
